# Should the Distal Splenorenal Shunt be Combined with Gastric Disconection and Transection?

**DOI:** 10.1155/1997/26507

**Published:** 1997

**Authors:** J. Michael Henderson

**Affiliations:** Department of General Surgery The Cleveland Clinic Foundation Cleveland Ohio 44195-5043 USA

## Abstract

*Background*: This study was aimed at evaluating advantages of distal splenorenal shunt (DSRS) with splenopancreatic and gastric disconnection (DSRS-SPGD) over DSRS with splenopancreatic disconnection (DSRS-SPD) and standard DSRS (S-DSRS).

*Methods*: DSRS-SPGD, DSRS-SPD, and S-DSRS were performed on 62, 7, and 55 patients, respectively, from 1970 to 1992. Comparison was performed in the following aspects: (1) long-term results in ratio of rebleeding, survival rate, and quality of life and (2) portal hemodynamics evaluated by preoperative and postoperative angiography. Portal blood flow was assessed by the ratio of the diameter of portal vein (PV) to superior mesenteric vein (SMV), and shunt selectivity was evaluated by selectivity grade.

*Results*: Incidence of rebleeding was significantly lower in patients who underwent DSRS-SPGD than in those who underwent S-DSRS (*p*< 0.05). Grade 0 and I performance status was better in patients who underwent DSRS-SPGD. Accumulated survival ratio for 5 and 7 years was 78.3% and 70.5% in patients who underwent DSRS-SPGD, 59.7% and 44.1% in patients who underwent S-DSRS, and 75% and 75% in patients who underwent DSRS-SPD. Hemodynamic evaluation showed significantly lower PV/SMV ratio and degree of change in PV/SMV ratio of patients who underwent S-DSRS and DSRS-SPD. Many patients who underwent S-DSRS and DSRS-SPD exhibited loss of shunt selectivity at grades II and III. In contrast, patients who underwent DSRS-SPGD maintained satisfactory PV/SMV ratio and selectivity grade.

*Conclusions*: DSRS-SPGD clearly showed advantages in decrease of rebleeding and improvement of quality of life resulting from maintenance of shunt selectivity and portal blood flow.


### SHOULD THE DISTAL SPLENORENAL SHUNT BE COMBINED WITH GASTRIC DISCONECTION AND TRANSECTION?

### ABSTRACT

*Kanaya, So-i. and Katoh, H. (1995) Long-term evaluation of distal splenorenal shunt with splenopancreatic and gastic disconnection. Surgery; 118: 29–35.*

***Background:*** **This study was aimed at evaluating advantages of distal splenorenal shunt (DSRS) with splenopancreatic and gastric disconnection (DSRS-SPGD) over DSRS with splenopancreatic disconnection (DSRS-SPD) and standard DSRS (S-DSRS).**
***Methods*****: DSRS-SPGD, DSRS-SPD, and S-DSRS were performed on 62, 7, and 55 patients, respectively, from 1970 to 1992. Comparison was performed in the following aspects: (1) long-term results in ratio of rebleeding, survival rate, and quality of life and (2) portal hemodynamics evaluated by preoperative and postoperative angiography. Portal blood flow was assessed by the ratio of the diameter of portal vein (PV) to superior mesenteric vein (SMV), and shunt selectivity was evaluated by selectivity grade.**
***Results:*** **Incidence of rebleeding was significantly lower in patients who underwent DSRS-SPGD than in those who underwent S-DSRS ($p<0.05$). Grade 0 and I performance status was better in patients who underwent DSRS-SPGD. Accumulated survival ratio for 5 and 7 years was 78.3% and 70.5% in patients who underwent DSRS-SPGD, 59.7% and 44.1% in patients who underwent S-DSRS, and 75% and 75% in patients who underwent DSRS-SPD. Hemodynamic evaluation showed significantly lower PV/SMV ratio and degree of change in PV/SMV ratio of patients who underwent S-DSRS and DSRS-SPD. Many patients who underwent S-DSRS and DSRS-SPD exhibited loss of shunt selectivity at grades II and III. In contrast, patients who underwent DSRS-SPGD maintained satisfactory PV/SMV ratio and selectivity grade.**
***Conclusions:*** **DSRS-SPGD clearly showed advantages in decrease of rebleeding and improvement of quality of life resulting from maintenance of shunt selectivity and portal blood flow. (Surgery 1995; *118*:29–35.)** *From the Second Department of Surgery, Hokkaido University School of Medicine, Sapporo, Japan*

KEY WORDS: Distal spenorenal shunt gastric disconnection and transection portal hypertension bleeding oesophageal varices.

### PAPER DISCUSSION

This paper[1] details long-term results from Kanaya and Katoh of a modification of selective variceal decompression that they have advocated in serial publications since 1988[2,3]. The concept of their work builds on the original idea behind selective shunts, namely, how to effectively decompress gastroesophageal varices while at the same time maintaining portal perfusion of the cirrhotic liver and thus liver function. Having learned early that variceal bleeding is well controlled with distal splenorenal shunt (DSRS), later work has focused on "disconnection" of the shunt and the portal hypertensive portal vein. What data are there on disconnection with DSRS, and how extensive should this be?

Warren's original paper on DSRS recognized the need for "portal azygos" disconnection as a component of this operation[4]. Interruption of the gastroepiploic arcade and ligation of the left gastric (coronary) vein were the components of this phase of the operation. Later it became recognized that there were three main routes of collateralization from the high pressure portal vein to the low pressure (shunted) splenic vein: pancreatic, gastric and mesocolic collaterals[5]. Concurrent with recognition of these pathways, it was also appreciated that while most patients will develop some or all of these collaterals, it was only in approximately 50% of patients with alcoholic cirrhosis that all prograde portal flow was lost through these collaterals[5,6]. How much portal flow must be maintained? This question has never been answered, mainly because there are other important factors, such as progression of the liver disease with ongoing viral replication or active alcoholism, which are often more important. For the patient with established alcoholic cirrhosis there is evidence that a more aggressive disconnection with DSRS is beneficial to such patients[7].

The mesocolic collateral pathway[5] – which developed large varices along the mesocolon to enter the splenic vein through the splenocolic ligament – is not seen when the splenicflexure of the colon is taken down at the primary operation. This should be done routinely, for in addition to the above, this operative move also greatly enhances exposure for dissection of the splenic vein and performance of the shunt.

The pancreatic siphon[5,8] after standard DSRS appeared to be the most critical path for loss of portal perfusion in alcoholic[5]. Data have documented that splenopancreatic disconnection – taking the entire splenic vein out of the pancreas – prevents this siphon, improves maintenance of portal perfusion in alcoholic patients to 84%, and improves their survival[7].

The transgastric collateral path has been recognized both primarily[5] but also as a secondary problem after DSRS with splenopancreatic disconnection[7]. The issue here is slightly different in that this pathway can give rise to recurrent gastric variceal bleeding[1,7], and is not usually associated with loss of all portal perfusion[5]. While some have dealt with this path by transhepatic embolization or reoperation to devascularize the lesser curve of the stomach[7], the approach advocated in the paper under discussion is appealing[1]. The primary gastric disconnection and transection may at first sight appear an overly aggressive procedure, but it makes physiologic sense, and as such warrants consideration. As originally recognized by Warren, Zeppa and Foman[4], there needs to be a portalazygos disconnection. The gastric disconnection advocated is an aggressive version of this. It has previously been recognized by esophageal[9] and gastric[10] "transectors" that varices will soon develop in the submucosal planes if there is no transection to a devascularization operation – hence there is established rationale for this component of the procedure.

Should all patients having DSRS have this extent of disconnection? Probably not. The primary goal is to achieve control of variceal bleeding – so the main aim has to be to get a good shunt to decompress the gastroesophageal varices. Many patients, particularly those with nonalcoholic disease, maintain good portal flow after standard DSRS, even if they develop some collaterals. The current paper is the only paper which shows this is beneficial to this group of patients. This author is not convinced that this extensive disconnection is necessary for this group of patients. For patients with alcoholic cirrhosis, the data in this [1] and other papers[7] support the use of extensive disconnection after DSRS to better maintain portal flow and liver function. Adding this gastric disconnection and transection makes sense for this subset of patients.

### REFERENCES

1. Kanaya, So-I. and Katoh, H. (1995) Long-term evaluation of distal splenorenal shunt with splenopancreatic and gastric disconection. *Surgery*, **118**, 29–35.
2. Katoch, H., Shimozawa, E. and Tanabe, T. (1985) Superselective distal splenorenal shunt for portal hypertension. *Gastroenterol Surg.*, **8**, 246–8.
3. Katoh, H., Shimozawa, E. and Kojima, T. *et al.* (1980) Modified splenorenal shunt with splenopancreatic disconnection. *Surgery*, **106**, 920–4.
4. Warren, W.D., Zeppa, R. and Foman, J.J. (1967) Selective transsplenic decompression of gastroesophageal varices by distal splenorenal shunt. *Ann Surg.*, **166**, 437–55.
5. Henderson, J.M., Gong-Liang, J., Galloway, J., Millikan, W.J. Jr., Sones, P.J. and Warren, W.D. (1985) Portaprival collaterals following distal splenorenal shunt: incidence, magnitude and associated portal perfusion changes. *J of Hepatol.*, **1**, 649–661.
6. Henderson, J.M., Millikan, W.J. Jr, Wright-Bacon, L, Kutner, M.H. and Warren, W.D. (1983) Hemodynamic differences between alcoholic and non-alcoholic cirrhotics following distal splenorenal shunt-effect on survival? *Annals of Surgery*, **198**, 325–334.
7. Henderson, J.M., Warren, W.D. and Millikan, W.J., *et al.* (1989). Distal splenorenal shunt with splenopancreatic disconnection: a 4-year assessment. *Ann Surg.*, **210**, 332–41.
8. Warren, W.D., Millikan, W.J. and Henderson, J.M., *et al.* (1986) Splenopancreatic disconnection: improved selectivity of distal splenorenal shunt. *Ann Surg.*, **204**, 346–55.
9. Crile, G. Jr. 1950 Transesophageal ligation of bleeding esophageal varices. *Arch Surg.*, **61**, 654–660.
10. Tanner, N.C. (1961) The late results of porto-azygosdiscon nection in the treatment of bleeding from oesophageal varices. *Annals of the Royal College of Surgeons*, **28**, 153–174.

J Michael Henderson, MD
Department of General Surgery
The Cleveland Clinic Foundation
Cleveland
Ohio 44195-5043
United States of America

